# Transcriptome Analysis of Potato Infected with the Necrotrophic Pathogen *Alternaria solani*

**DOI:** 10.3390/plants10102212

**Published:** 2021-10-18

**Authors:** Sophie M. Brouwer, Maja Brus-Szkalej, Ganapathi V. Saripella, Dong Liang, Erland Liljeroth, Laura J. Grenville-Briggs

**Affiliations:** 1 Department of Plant Protection Biology, Swedish University of Agricultural Sciences, P.O. Box 7070, SE-750 07 Uppsala, Sweden; maja.brus@slu.se (M.B.-S.); Dong.Liang@slu.se (D.L.); Erland.Liljeroth@slu.se (E.L.); 2 Department of Plant Breeding, Swedish University of Agricultural Sciences, P.O. Box 7070, SE-750 07 Uppsala, Sweden; ganapathi.varma.saripella@slu.se; 3 Institute of Plant Protection, Jiangsu Academy of Agricultural Sciences, Nanjing 210014, China

**Keywords:** early blight, RNAseq, necrotroph, *Solanum tuberosum*, plant-pathogen interaction

## Abstract

Potato early blight is caused by the necrotrophic fungus *Alternaria solani* and can result in yield losses of up to 50% if left uncontrolled. At present, the disease is controlled by chemical fungicides, yet rapid development of fungicide resistance renders current control strategies unsustainable. On top of that, a lack of understanding of potato defences and the quantitative nature of resistance mechanisms against early blight hinders the development of more sustainable control methods. Necrotrophic pathogens, compared to biotrophs, pose an extra challenge to the plant, since common defence strategies to biotic stresses such as the hypersensitive response and programmed cell death are often beneficial for necrotrophs. With the aim of unravelling plant responses to both the early infection stages (i.e., before necrosis), such as appressorium formation and penetration, as well as to later responses to the onset of necrosis, we present here a transcriptome analysis of potato interactions with *A. solani* from 1 h after inoculation when the conidia have just commenced germination, to 48 h post inoculation when multiple cell necrosis has begun. Potato transcripts with putative functions related to biotic stress tolerance and defence against pathogens were upregulated, including a putative Nudix hydrolase that may play a role in defence against oxidative stress. *A. solani* transcripts encoding putative pathogenicity factors, such as cell wall degrading enzymes and metabolic processes that may be important for infection. We therefore identified the differential expression of several potato and *A. solani* transcripts that present a group of valuable candidates for further studies into their roles in immunity or disease development.

## 1. Introduction

Early blight of potato is a disease caused by fungi belonging to the genus of *Alternaria*. Even though several species of *Alternaria,* such as *A. alternata* and *A. grandis* have been indicated as the causal agent of potato early blight [1,2], *A. solani* is considered to be the main causal agent in Sweden [1]. *A. solani* is generally considered to be a necrotrophic fungus and the large-scale necrosis of infected tissues and subsequent defoliation, if left untreated, lead to the potential for early blight to halve potato yield [3]. The disease is currently controlled by fungicide treatment, however, fungicide resistance in *A. solani* populations has been reported for several of the commonly used fungicides [4]. Although cultivars with varying levels of early blight resistance exist, the resistance is suggested to be quantitative since no specific resistance (R) genes have been identified, despite extensive investigation. Resistance appears to be closely linked to the maturity of cultivars and exhibits quantitative inheritance and race-specific resistance has so far not been observed [5]. 

The *A. solani* infection process was analyzed in a histopathological study of three different cultivars with varying early blight resistance by Dita et al. [6]. This study found that *A. solani* forms appressoria to penetrate host tissue and that the cultivar with the highest level of early blight resistance displayed hypersensitive response (HR) like symptoms, such as a granular structure of the cytoplasm and a thickened cell wall in the epidermal cells where the appressoria were formed. Due to the necrotrophic lifestyle of *A. solani*, the positive correlation of HR like symptoms and early blight resistance is puzzling, since necrotrophic pathogens are generally considered to benefit from HR in establishing infection [7,8]. In a recent study, we showed that, in contrast with the generally reported larger role of jasmonic acid (JA) in the defense against necrotrophic plant pathogens, potato requires intact salicylic acid (SA) signaling for defenses against *A. solani* [9]. We found that SA deficient plants displayed increased lesion development and less H_2_O_2_ production. One possible interpretation of these data is that *A. solani* is in fact not a true necrotroph but a hemibiotroph with a very short biotrophic phase.

In our recent work, we used microarrays to analyze the transcriptional changes occurring upon infection with *A. solani* in the susceptible potato cultivar Désirée, and SA and JA deficient lines where the earliest time point of sampling was 24 h post inoculation [9]. In the current study, we continued our investigation of *A. solani* induced transcriptional changes in potato in more detail. We have now used RNA sequencing to study the transcriptional changes occurring in potato foliage due to infection with *A. solani* in the same potato cultivar as used in our previous study. Additionally, the use of RNA sequencing allowed for the analysis of transcriptional changes in *A. solani* during the infection process. We focused on the transcriptional changes of *A. solani* and *S. tuberosum* in, and near, the inoculated area, starting as early as 1 h post inoculation up to 48 h post inoculation. Since, the *A. solani*–potato interaction displays some characteristics generally not seen in necrotrophic plant–pathogen interactions, such as the formation of appressoria and an important role of intact SA signaling in the restriction of lesion development, the early time points were specifically included to allow for detection of transcriptional changes in *A. solani* and inoculated potato before necrosis occurs. We therefore aimed to obtain data to understand if *A. solani* behaves as a hemi-biotroph with a short biotrophic phase on potato, or acts more like a necrotroph even from the early onset of infection.

## 2. Results

### 2.1. Alternaria solani Infection Progress over a Time Course of 48 Hours

**Figure 1:**
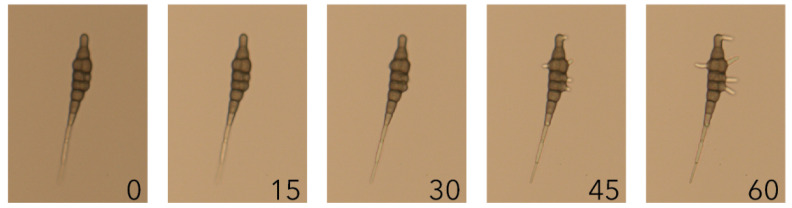
Germination of *A. solani* conidium. *A. solani* conidium in sterile tap watered imaged every 15 min for the first hour after harvesting. The numbers indicate minutes after harvest.

**Figure 2:**
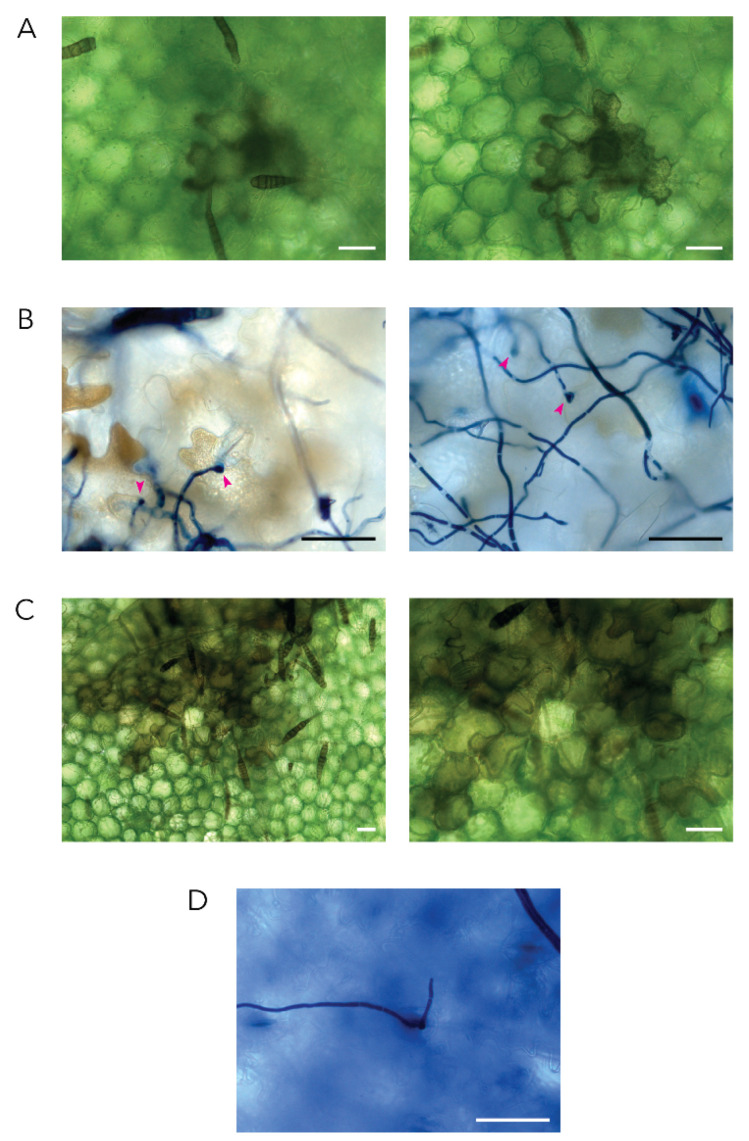
Infection of potato by *A. solani*. Representative micrographs of (**A**) death of a potato adaxial epidermal cell due to *A. solani* infection as observed in perfluorodecalin prepared samples harvested at 24 hpi. (**B**) Appressoria formed by *A. solani* to penetrate the adaxial side of the leaf at 24 hpi as observed in trypan blue stained samples harvested at 24 hpi. Appressoria are indicated with a pink arrow. (**C**) Adaxial epidermal and mesophyll cell death due to *A. solani* as observed in perfluorodecalin prepared samples harvested 48 hpi. (**D**) Emergence of *A. solani* hyphae through a stoma on the abaxial side of the leaf as observed in trypan blue stained samples harvested at 48 hpi. Scale bars in all images correspond to 50 µm.

In this study *Alternaria solani* conidia were used as inoculum. The *A. solani* conidia are claviform in shape (i.e., shaped like clubs or baseball bats) and can form multiple germ tubes (Figure 1). Germination of the conidia occurs under high humidity conditions, independent of the presence of a host. Conidia, both in vitro and in the inoculation droplet on a potato leaf, were observed to germinate within one hour (Figure 1). Six hours post inoculation (hpi) on the potato leaf, further growth of the germ tubes both towards and away from the plant epidermal cell surface occurred. At 12 hpi the germ tubes had formed a network of fungal growth within the inoculation droplet, and the first formation of appressoria and penetration had occurred. After 24 hpi, necrosis of the first epidermal cells on the adaxial side of the leaf due to *A. solani* infection was observed (Figure 2A), and the presence of appressoria was observed consistently (Figure 2B). Host cell death increased, and at 48 hpi the necrosis of groups of multiple epidermal cells and mesophyll cells was observed (Figure 2C). Additionally, emergence of hyphae through the stomata on the abaxial side of the leaf was observed (Figure 2D). 

### 2.2. Mapping of Reads to Potato and A. solani Reference Genomes

The response of potato gene expression to infection with *A. solani* and the changes occurring in *A. solani* gene expression during the infection of potato were analyzed using RNA sequencing. Drop inoculated samples were harvested at 1, 6, 12, 24, and 48 hours post inoculation (hpi). Three biological replicates were used for each sample. Mapping of the reads was performed first to the potato reference genome and subsequently to the *A. solani* reference genome. The total number of reads, the percentage of reads mapping to the *S. tuberosum* genome and the percentage of *S. tuberosum* unmapped reads mapping to the *A. solani* genome are presented in Table 1. Due to the lack of transcript annotations for the *A. solani* genome, annotations were generated using the microbial fast annotation tool RASTtk and by NCBI BLAST analysis against *A. alternata* [10]. 

### 2.3. Differential Expression of Plant Transcripts during Infection

**Figure 3:**
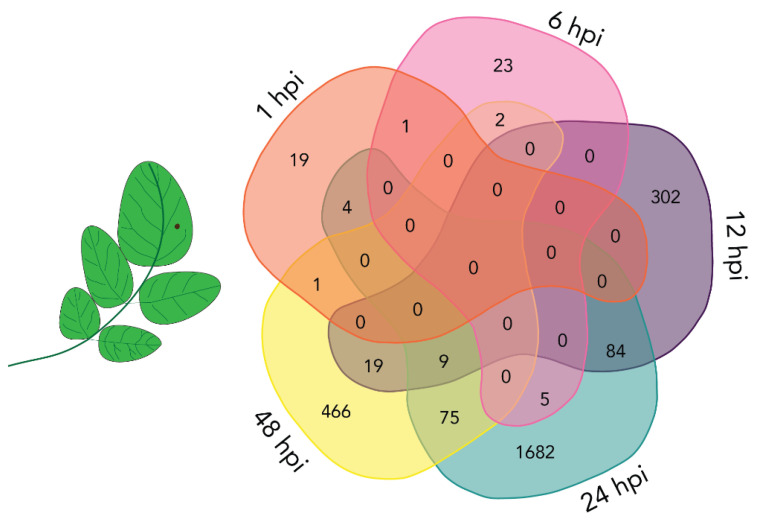
Venn diagram displaying overlap between differentially expressed transcripts (DETs) from potato detected at different time points after inoculation (hours post inoculation, hpi). Comparison between DETs at 1 hpi (orange), 6 hpi (pink), 12 hpi (purple), 24 hpi (green) and 48 hpi (yellow). Numbers represent unique or overlapping DETs between time points.

The differential plant transcript expression analyses were performed for the *A. solani* versus mock inoculated samples at the five time points. Transcripts were considered to be differentially expressed when the adjusted *p*-value was < 0.05. No log2 fold change cut-off was used. The number of differentially expressed transcripts (DETs) from the host plant for all time points is presented in Figure 3, and the complete list can be found in the supplementary File S1. The number of DETs increased with time post inoculation. However, more DETs were found at 24 hpi compared to 48 hpi, which may reflect a lower level of overall gene expression due to necrotic and dying tissue at 48hpi. 

### 2.4. Mostly Unique Plant DETs at Different Time Points 

The overlap between *S. tuberosum* DETs at the five time points was analyzed (Figure 3) and the comparison revealed that none of the DETs were common to all five of the time points. The largest overlap in DETs was found between the latest three time points. However, the majority of the plant DETs for each time point were unique, indicating distinct plant responses to the progression from pathogen inoculation to cell death (Figure 3). 

### 2.5. Gene Ontology Enrichment Analysis 

**Figure 4:**
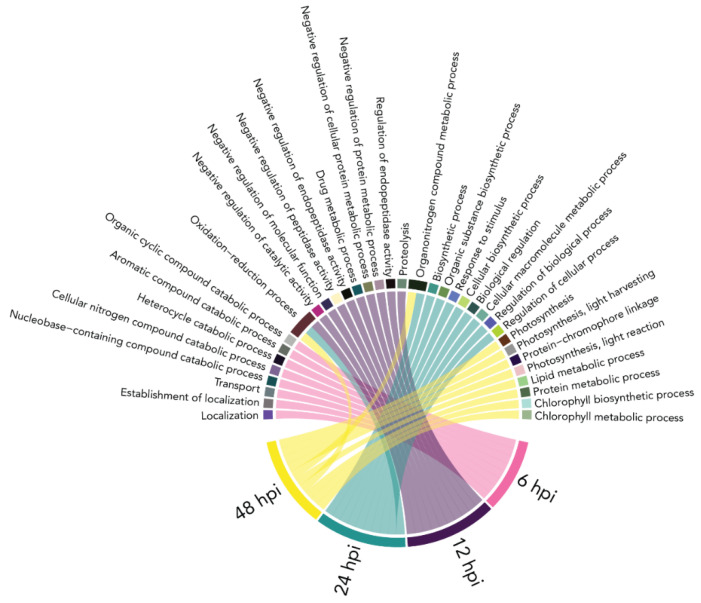
Gene ontology (GO) enrichment of differentially expressed plant transcripts at 6, 12, 24, and 48 h post A. solani inoculation (hpi). Chord diagram showing top 10 significantly enriched (FDR < 0.05) biological process GO-annotations for each time point.

In order to gain a better understanding of the function of the differentially expressed plant transcripts, a gene ontology (GO) enrichment analysis of the genes belonging to the DETs of the host plant, *Solanum tuberosum,* was performed. For the DETs at 1 hpi, no significantly enriched GO terms were found. For the other time points, several enriched GO terms were found. The top ten biological process GO terms for these time points and the overlap between them are visualized in Figure 4.

### 2.6. Differential Expression of Biotic Stress Related Plant Transcripts

**Figure 5:**
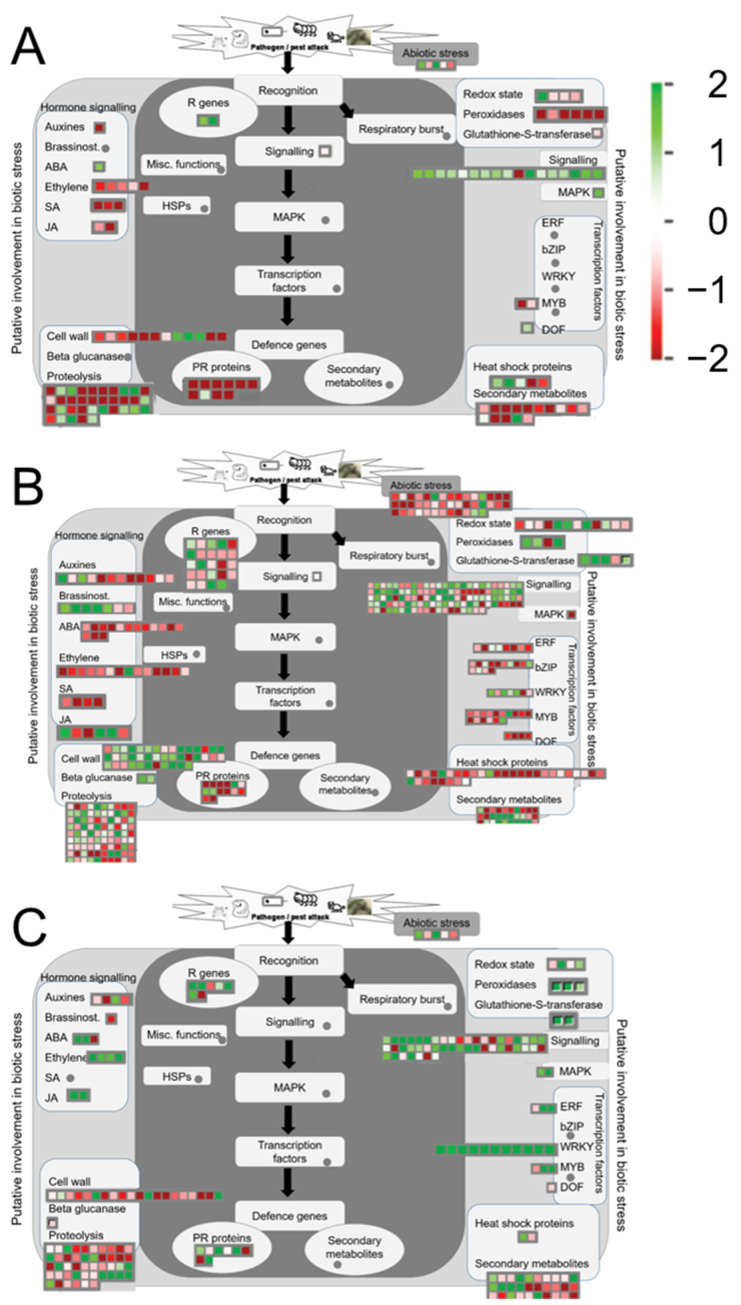
MapMan illustration of DETs belonging to the ‘Biotic stress’ BIN at (**A**) 12 hpi, (**B**) 24 hpi (**C**) 48 hpi. Log2 fold change is indicated as gradient from green (upregulated) to red (downregulated).

Further functional analysis of DETs at 12, 24 and 48 hpi was performed using MapMan software (version 3.6.0R1 https://mapman.gabipd.org/home, last accessed on 20 November 2020) with the *Solanum tuberosum* PGSC transcript mapping file exported from gomapman.org [11]. Differential expression of transcripts related to ‘biotic’ stress were detected at 12 hpi with upregulation of transcripts belonging to ‘R genes’ and ‘signalling’ bins. ‘Cell wall’ and ‘proteolysis’ transcripts showed both up- and downregulation. ‘PR’ proteins were mainly downregulated (Figure 5A). At 24 hpi, more transcripts related to biotic stress were differentially expressed, with ‘R genes’ ‘signalling’, ‘cell wall’, ‘proteolysis’, ‘transcription factors’, ‘hormone signalling’ and ‘heat shock’ transcripts showing differential expression (Figure 5B). At 48 hpi, in line with the general lower number of DETs compared to 24 hpi, the number of ‘biotic stress’ related transcripts was lower. At 24 hpi, downregulation of ‘biotic stress’ related ‘transcription factor’ transcripts was mainly observed, yet at 48 hpi, the transcripts in the ‘transcription factor’ bin showed mostly upregulation. Additionally, transcripts related to ‘hormone signalling’ showed more upregulation at 48 hpi compared to the earlier time points (Figure 5C).

### 2.7. Differential Expression of A. solani Transcripts during Infection 

**Figure 6:**
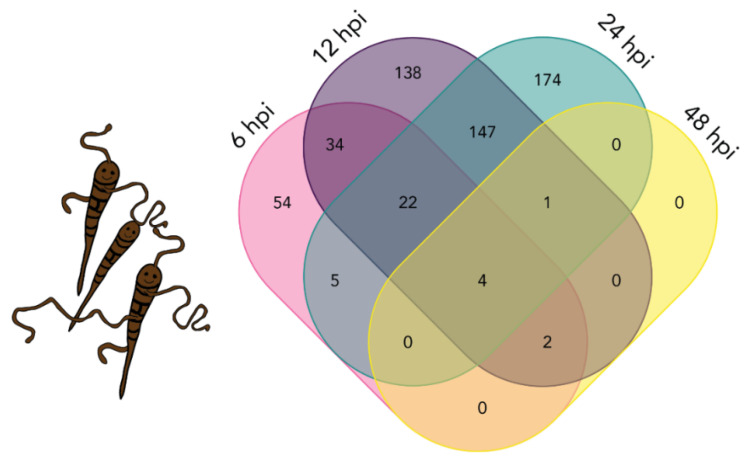
Venn diagram displaying overlap between the numbers of differentially expressed transcripts (DETs) from *A. solani* at different time points. Overlap between *A. solani* DETs at 6 hpi (pink), 12 hpi (purple), 24 hpi (green) and 48 hpi (yellow).

Differential *A. solani* transcript expression analyses were performed by comparing the *A. solani* inoculated samples at 6, 12, 24, and 48 hpi with the 1 hpi sample. The number of *A. solani* DETs is presented in Table 2, and the complete list is given in the supplementary File S2. The number of DETs increased with time, with the exception of 48 hpi, when the fewest DETs were detected (Figure 6). The overlap between *A. solani* DETs at the four time points was analyzed (Figure 6). The *A. solani* DETs at the different time points show more overlap than the potato DETs. The largest overlap was observed between two consecutive time points. Notably, of the seven DETs observed for the last time point, the majority of the genes were also differentially expressed in all other time points (Figure 6). 

### 2.8. Quantitative Real Time PCR Gene Expression Analysis Correlates Well with RNA Sequencing Data 

The differential expression of the selected top *A. solani* transcripts, found both across all of the different sampling time points (Table 3) and at specific time points only (Table 4), was confirmed by quantitative RT-PCR (qRT-PCR) performed on independent biological replicates. A Pearson correlation test showed a significant correlation between the RNA sequencing and qRT-PCR data sets with an R2 value of 0.9162 and a correlation coefficient of 0.9572 (*p* value < 0.0001), validating the RNA sequencing data. A linear regression of the correlation is plotted in supplementary Figure S1. 

## 3. Discussion

### 3.1. Differential Expression of Solanum tuberosum Transcripts

At 1 and 6 h post inoculation, the *A. solani* conidia had started germination, but no formation of appressoria or penetration had occurred. However, for both time points, we detected DETs for *S. tuberosum* (supplementary File S1). At 1 hpi, downregulation of a gene annotated as stress regulated protein was observed (Table 2). This gene was shown to be upregulated by the synthetic SA homolog benzothiadiazole (BTH), but downregulated by the plant resistance inducer β-aminobutyric acid (BABA) (Results from AN Massa, KL Childs, H Lin, GJ Bryan, G Giuliano and CR Buell [13], visualized in Potato eFP browser by D Winter, B Vinegar, H Nahal, R Ammar, GV Wilson and NJ Provart [14]). One of the most highly upregulated transcripts at 1 hpi encoded a Mutt domain protein (Table 2). This transcript was also differentially expressed at 48 hpi, but at this time point showing downregulation (log2 FC −7.76) (supplementary File S1). The Mutt motif is also called the nucleoside diphosphates linked to some moiety X (Nudix) box. The closest *Arabidopsis thaliana* protein match to the potato Mutt domain protein was cytosolic Nudix hydrolase AtNUDX2, encoded by At5g47650. Overexpression of this gene in *A. thaliana* was shown to increase tolerance to oxidative stress [15]. Additionally, other cytosolic *A. thaliana* Nudix hydroxylases have been shown to play both positive and negative roles in biotic stress responses [16,17,18]. AtNUDX7 was shown to be a negative regulator of the EDS1 signaling pathway, a key component of plant immunity regulation. Knockout mutants of AtNUDX7 accumulated higher levels of SA than the control, showed constitutive expression of defense genes and had enhanced resistance against both the oomycete *Hyaloperonospora arabidopsidis* and the bacterium *Pseudomonas syringae* pv. *maculicola* [19]. AtNUDX8, however, was shown to positively regulate immunity with knockout mutants showing repressed SA signaling and enhanced susceptibility to *H. arabidopsidis* and *P. syringae* pv. *maculicola* [18]. In summary, the Nudix proteins in *A. thaliana* play important roles in plant defenses and can influence the levels of SA. Further research into the potato Mutt domain protein and its role in susceptibility or resistance to *A. solani* is suggested. Among the plant DETs at 6 hpi is a transcript encoding Sn-2 protein (PGSC0003DMT400006208) (supplementary File S1). The encoded protein shares 89% protein similarity with a putative PR-10 type pathogenesis-related protein in *Nicotiana tabacum*. Downregulation of this gene was also observed in pooled data from 24, 36 and 72 hpi of potato infected with the late blight pathogen *Phytophthora infestans* (Results from AN Massa, KL Childs, H Lin, GJ Bryan, G Giuliano and CR Buell [13], visualized in Potato eFP browser by D Winter, B Vinegar, H Nahal, R Ammar, GV Wilson and NJ Provart [14]).

### 3.2. Gene Ontology Enrichment of Drug Metabolic Processes at 12 hpi 

Gene ontology (GO) analysis of the plant differentially expressed transcripts was performed and at 12 hpi the GO term ‘drug metabolic process’ was significantly enriched. One of the ‘drug metabolic process’ associated transcripts showing upregulation encodes pectinesterase 3. Pectinestarase, also called pectinmethylesterase, catalyzes the removal of methylesther groups from pectin [20]. The plant cell wall contains pectic polysaccharides and the degradation of the cell wall by necrotrophic plant pathogens, including pectin degradation, is required to release nutrients [21]. However, induction of plant pectinestarases during infection has also been observed. In *Aradidopsis thaliana*, a significant increase in gene expression of pectin methylesterase AtPME3 (At3g14310) was observed after inoculation with the necrotrophic fungus *Botrytis cinerea* and bacterium *Pectobacterium carotovorum*. AtPME3 was shown to be a susceptibility factor for infection by these pathogens, since pectin methylestarase mutant plants showed reduced susceptibility compared to the wild type [22]. The potato pectinestarase transcript we observed as differentially expressed upon inoculation with *A. solani* presents an interesting target for further study.

### 3.3. Gene Ontology Enrichment of Photosynthesis Related Plant Transcripts at 48 hpi

At 48 hpi, biological process GO terms associated to photosynthesis were significantly enriched (Figure 6). All transcripts belonging to the genes enriched in the photosynthesis GO terms were downregulated. More than half of these genes are annotated as a chlorophyll a–b binding protein that is a part of the light harvesting complex of chlorophyll. Downregulation of genes involved in photosynthesis can either be an energy preserving strategy to invest in upregulation of defense genes, or a reaction to the large increase of *A. solani*-induced necrosis. Previously, a comparison of 22 different biotic stress interactions with eight different plant species revealed that the downregulation of photosynthesis genes was a global response to biotic stress [23]. 

### 3.4. Differential Expression of Hormone Biosynthesis Related Transcripts during A. solani Infection 

Ethylene and jasmonic acid signaling are generally considered to be required for activation of defenses against necrotrophic plant pathogens [24]. However, interestingly, at 12 hpi we observed downregulation of a transcript encoding 1-aminocyclopropane carboxylic acid oxidase 2 (PGSC0003DMT400036081) (supplementary File S1, an enzyme involved in the last step of ethylene biosynthesis [25]. At 24 hpi, this transcript was still downregulated, together with two other 1-aminocyclopropane carboxylic acid oxidase encoding transcripts (PGSC0003DMT400043087 and PGSC0003DMT40004444) (supplementary File S1). Similarly, transcripts for the JA biosynthesis involved enzyme lipoxygenase PGSC0003DMT400081909 at 12 hpi and PGSC0003DMT400058933 at 24 hpi, and allene oxide synthase (PGSC0003DMT400027377) were downregulated at 12 hpi and 24 hpi (supplementary File S1) However, other lipoxygenase transcripts (PGSC0003DMT400063468 and PGSC0003DMT400028158) showed upregulation at 24 hpi and 48 hpi, respectively (supplementary File S1). Interestingly, at 12 hpi, three SA signaling transcripts all encoding salicylic acid carboxyl methyltransferases, were downregulated. The same transcripts were still downregulated at 24 hpi. Overexpression of a SA carboxyl methyltransferase gene from rice in *A. thaliana* rendered the plants more susceptible to infection by the hemibiotroph *P. syringae* and the obligate biotrophic fungus *Golovinomyces orontii* [26]. We previously showed that intact SA signaling is required for potato defenses against *A. solani,* since plants deficient in SA accumulation developed larger lesions [9]. This, together with the downregulation of ethylene and jasmonic acid biosynthesis genes observed at the early time points of *A. solani*, indicates that *A. solani* does not trigger responses characteristic for defense against necrotrophs in potato during the initial stages of infection. 

### 3.5. A. solani DETs Overlapping in all Four Time Points 

The RNA sequencing analysis revealed four *A.solani* DETs overlapping in all four time points, of which two transcripts were downregulated and two were upregulated in all time points compared to 1 hpi (Table 3). One of the downregulated transcripts encodes a putative pectate lyase (Table 3). In the related fungus, *Alternaria brassicicola*, a pectate lyase encoding gene *PL1332* was shown to be highly expressed up to 12 hpi and was shown to be required for full virulence. Additionally, potato apoplast injection with a fusion protein of PL1332 resulted in necrosis of the plant tissue, indicating a cell wall degrading function [27]. In our analysis, we found the pectate lyase transcript was downregulated at 6, 12, 24, and 48 hpi compared to 1 hpi, indicating a possible role of the enzyme early on at the start of the germination phase in the presence of the plant. Additionally, the encoded protein is predicted to contain a signal peptide, and InterPro analysis predicts the protein to by non-cytoplasmic (Table 4). Another transcript upregulated in all time points encodes a NADP- dependent mannitol dehydrogenase (Table 3). NADP-dependent mannitol dehydrogenases catalyze the conversion of fructose into mannitol. Mannitol biosynthesis was shown to play a role in pathogenicity of *A. alternata* and *A. brassicicola*, but was not required for germination of conidia [28,29]. Moreover, pathogen mannitol has been shown to interfere with the formation of physical barriers in the plant host and to scavenge reactive oxygen species (ROS) [30]. 

### 3.6. A. solani DETs Reveal Potential Pathogenicity Factors

The most upregulated transcript (mRNA_9018) at both 6 and 12 hpi, encodes an aldehyde dehydrogenase (Table 4). This transcript was still found to be upregulated at 24 hpi (log2FC = 8.07) (supplementary File S2). Aldehyde dehydrogenases (ALDHs) are evolutionarily conserved enzymes employed for reactive molecule scavenging and are found in both plants and pathogens. ALDHs can also function as allergens, such as Alt a 10 from *A. alternata* (EC 1.2.1.3 ALDH) a causal agent of human allergies [31]. However, ALDHs were also shown to play a role in the pathogenicity of plant pathogens. RNA interference (RNAi) silencing of two ALDHs in the rice blast fungus *Magnaporthe oryzae* resulted in reduced conidiogenesis, vegetative growth and pathogenicity, the mutants were also highly sensitive to several oxidative and reductive stress inducing agents. These data indicate the importance of the ALDHs in tolerance of *M. oryzae* to reactive oxygen species (ROS) and reactive aldehydes generated by both the plant and pathogen during the infection process [32]. 

At 6 hpi compared to 1 hpi we observed downregulation of a transcript (mRNA_9891) predicted to encode a flavin-dependent monooxygenase (Table 4). This same transcript was also differentially expressed at 12 hpi (log2FC = −5.36) (supplementary File S2). Flavin-dependent monooxygenases catalyze a wide variety of oxygenation reactions and are found in many microorganisms. The gene *AbMak1* encoding a flavin-dependent monooxygase in *A. brassicicola* was shown to be important for melanin production and conidial cell wall structure, since a lower melanin content and an aberrant cell wall were observed in Δ*abmak1* mutants. The mutants were, however, not affected in pathogenicity and susceptibility to various stress conditions [33], indicating that this flavin-dependent monooxygenase in *A. brassicicola* is important for conidia structure. In *A. solani*, the conidia are highly melanized, yet the germ tubes are less melanized (Figure 1). If the encoded mRNA_9891 monooxygenase is important for melanization, downregulation during germination might be expected, which is what we observed. At 12 hpi, formation of the first appressoria was observed. The second highest upregulated transcript (mRNA_29) is predicted to encode a trehalase, catalyzing the conversion of trehalose to glucose. The transcript was still found to be upregulated at 24 hpi (log2 FC = 7.04) (supplementary File S2). The encoded protein sequence is predicted to contain a signal peptide and non-cytoplasmic domain, and is thus likely secreted (Table 4). Trehalose is a disaccharide that has been shown to confer protection against various environmental stresses and is found in both plants and fungi [34]. In *M. oryzae*, both the synthesis of trehalose by a trehalose-6-phosphate synthase and the breakdown of trehalose by trehalase were shown to play a role in the infection of rice. The synthase was shown to be required for appressorium-mediated penetration and the trehalase for the development of infection after penetration. In summary, trehalose metabolism appears to play an important role in the pathogenicity of *M. oryzae* and our data suggests it may also be important for *A. solani* pathogenicity as well. 

At 24 hpi, upregulation of mRNA_2512, annotated as encoding an oxidoreductase belonging to the glucose-methanol-choline (GMC) family, was seen (Table 4). This transcript also showed upregulation at 12 hpi (log2 FC = 6.85) (supplementary File S2). The protein encoded by this transcript is predicted to contain a signal peptide and a non-cytoplasmic domain (Table 4). Characterized members of the GMC enzyme family from fungi have been shown to be relevant for lignocellulose degradation [35]. The encoded enzyme of mRNA_2512, therefore, might play a role in the degradation of the plant cell wall. At 24 hpi, compared to 1 hpi, downregulation of mRNA_9110 (predicted to be an S-adenosyl-L-homocysteine hydrolase (SAHH)) was observed (Table 4). This transcript was also downregulated at 12 hpi (log2 FC = −3.67) (supplementary File S2). The enzyme SAHH is an important enzyme in the methylation potential in the cell. SAHH catalyzes the reversible hydrolysis of S-adenosylhomocysteine (SAH) to adenosine and homocysteine. SAH is converted into the major methyl donor S-adenosylmethionine (SAM). SAHH have been shown to be important in plant responses to pathogens in several plants [36]. However, SAHH has also been shown to have a role in fungal pathogenicity, e.g. in the chestnut blight fungus *Cryphonectria parasitica.* SAHH knockout mutants showed a reduced growth rate, absence of conidia and reduced virulence with reduced expression of virulence genes. We hypothesized that since SAHH is a key enzyme required for the methylation potential of the cell, the Δ*sahh* mutants have altered DNA methylation, which results in altered gene expression. However, the exact role of SAHH in *C. parasitica* virulence remains to be elucidated [37]. For the hypothetical protein transcripts mRNA_11966, mRNA_9008 and mRNA_8569, signal peptides and non-cytoplasmic domains were predicted, however further InterPro analysis did not predict any protein family membership. Their differential expression and indication of secretion make them interesting targets for validation studies of their role in pathogenicity as potential effector proteins. Fungal effector proteins are generally small (<200 amino acids) cysteine-rich (2–20%) secreted proteins [38]. The predicted protein sequence of mRNA_8569 is 109 amino acids long and has a cysteine content of 3.9%. Functional validation of mRNA_8569 to determine whether the encoded protein operates as an effector would thus be of particular interest. 

## 4. Materials and Methods

### 4.1. Plant Material and Growth Conditions

In Vitro maintained *Solanum tuberosum* cultivar Désirée was grown in 2 L pots containing commercial soil (Exclusiv Blom & Plantjord, Emmaljunga Torvmull AB, Sweden) supplemented with 15 mL of fertilizer beads (Osmocote exact 3–4 months, ICL, Ipswich, UK). The plants were placed in an artificial light chamber with 14 h of 160 µmol/s/m^2^, 65% relative humidity (RH) at 20 °C. To allow acclimatization, the plantlets were covered with plastic cups during the first week in soil. 

### 4.2. Alternaria solani Maintenance and Inoculum Preparation

*Alternaria solani* strain NL03003 (CBS 143772) [39] was maintained on V8 solid medium. The plates were kept in the dark at room temperature for 4 days, and subsequently placed in an 18 °C incubator equipped with UV-c light bulbs (model OSRAM HNS15G13 with dominant wavelength 254 nm) supplying 8 h of UV-c light per day for 10 days to induce sporulation. Conidia for inoculations were harvested by flooding the plates with 10 mL sterile tap water and gently rubbing the mycelium to dislodge the conidia using plastic L-shaped spatulas and slow pipetting up and down. The concentration of conidia was determined using a Fuchs Rosenthal haemocytometer. For all infections, an inoculum of 25,000 conidia/mL was prepared. 

### 4.3. Microscopy

Potato leaf punches inoculated with *A. solani* NL3030 were placed onto microscopy slides either in perfluorodecalin infiltrative imaging medium for improved visualization [40] or stained with trypan blue according to the method previously described [41]. Bright field microscopy of leaf punches and *A. solani* conidia in sterile tap water was performed using an inverted Zeiss Axio Observer D1. Micrographs were processed in ZEN 3.1 (blue edition) (Zeiss, Germany). 

### 4.4. Plant Inoculation with Alternaria solani

Six-week old potato plants were arranged on trolleys containing 6 plants each. The trolleys were covered in plastic foil to reach >95% RH and placed inside an artificial light plant chamber at 20 °C and 90% RH, receiving 14 h of 160 µmol/s/m^2^ light. Per treatment, 3 trolleys were prepared, from which one plant per trolley was sampled for each time point. 6 leaflets per plant were inoculated on the adaxial side of the leaf with either 10 µL droplets containing 25,000 conidia/mL *A. solani* NL03003, or mock inoculum. The plants were inoculated right before the lights turned off, and the trolleys were covered with plastic foil for the first 24 h to ensure the high humidity required for the start of infection. Leaf disc samples were taken including the inoculation spot using an 8 mm cork borer, collecting 5 leaf discs from the same plant in one 1.5 mL microcentrifuge tube snap-frozen in liquid nitrogen. Samples were collected at 1, 6, 12, 24, and 48 h post inoculation (hpi), immediately snap-frozen, and ground in liquid nitrogen prior to RNA extraction.

### 4.5. RNA Preparation and Sequencing

RNA preparation and sequencing was performed as described previously [42]. Briefly, approximately 100 mg of plant material (5 leaf discs) was used for RNA extraction using the Qiagen RNeasy Plant Mini kit (Qiagen, Hilden, Germany), according to the manufacturer’s protocol, with an added DNase treatment interruption step. The DNase treatment was performed on column using the Invitrogen PureLink™ DNase set (Thermo Fisher Scientific, Massachusetts, USA) according to the manufacturer’s protocol. The RNA quality and concentration were both corroborated by ND-1000 NanoDrop and by a 2100 Bioanalyzer using RNA Nano chips (Agilent Technologies, CA, USA). Polyadenylated messenger RNA was captured from 200 ng total RNA per sample using magnetic beads and Illumina adaptors with sample-specific barcode sequences that were ligated before subsequent library amplification using PCR via the Illumina TruSeq RNA poly-A selection kit. Sequencing of 150 bp paired-end libraries was carried out using the Illumina NovaSeq6000 S4 platform (SciLifeLab, Stockholm, Sweden). All raw sequencing data in this study have been deposited in National Center for Biotechnology Information (NCBI) under BioProject accession number PRJNA755645.

### 4.6. Expression Analysis from RNA Sequencing

Initial quality control (QC) of the paired-end mRNA reads generated using Illumina high-throughput sequencing was performed at the NGI facility, Stockholm. An initial filtering step was performed for removal of ribosomal RNAs (rRNAs) by aligning reads with the silva and rfam databases using the Sortmerna-v2.1b [43] tool, and all TruSeq3 adapters were trimmed with Trimmomatic-v0.36 [44], setting MINLEN:20 in bases and SLIDINGWINDOW:5:20 with other parameters being the defaults. A second round QC check was performed on independent samples with FastQC v0.11.7 [45] and the multiple sample visualization MultiQC v1.6 [46] tool was used. The whole genome of PGSC_v4.03 (The Potato Genome Sequencing Consortium et al., 2011) was used for reference alignment. The mRNA reads were aligned to the genome using splice aligner STAR-v2.5.4a [47] tool with, --twopassMode Basic, --sjdbGTFfeatureExon CDS, --outReadsUnmapped Fastx, keeping other parameters as default. Transcript abundance was estimated with Salmon v1.3.0 [48]. Raw read counts were used for differential expression (DE) analysis with DESeq2 [49,50] and the built-in cross sample relative log expression” (RLE) [51] normalization was performed. The unmapped reads from the potato genome map were further processed and mapped to the *Alternaria solani* genome BMP0185 [52] using the same standardized methods for mapping, quantification and DE described above. The annotation to the *A. solani* genome was adopted and performed with fast annotation search tool RAStk [10]. In addition, BLAST searches (ncbi-blast-v2.9.0) with -max_target_seqs 1, -evalue 1e-5 were performed against transcripts of the closely related species *Alternaria alternata* for comparative analysis. Plant DE analysis was performed comparing the *A. solani* inoculated samples with the mock inoculated samples at the same time point. *A. solani* DE analysis was performed comparing all later time points individually with the first time point, 1 hpi. Transcripts were considered to be differentially expressed when the adjusted *p*-value < 0.05. No log2 fold change cut-off was used. Venn diagrams were created using an online tool (http://bioinformatics.psb.ugent.be/webtools/Venn/, last accessed on 5 November 2020). The gene ontology (GO) enrichment analysis was performed using ShinyGO v0.61 to detect significantly enriched GO terms (FDR < 0.05) using the default settings, with *Solanum tuberosum* as the matched species [53]. The top-10 enriched biological process GO terms were visualized in a chord diagram created using the R package circlize [54]. Additional functional analysis was performed in MapMan (version 3.6.0R1 https://mapman.gabipd.org/home, last accessed on 20 November 2020) using the *Solanum tuberosum* PGSC transcript mapping file exported from gomapman.org [11]. Prediction of signal peptides from *A. solani* transcripts was performed by translating the mRNA nucleotide sequence into amino acids using EMBOSS Transeq [55], followed by eukaryote signal peptide prediction using SignalP 5.0 [56]. Sequences with a likelihood of >0.9 were considered to contain a predicted signal peptide. InterPro [12] was used to predict the presence of a non-cytoplasmic domain. The cysteine content of predicted proteins was performed using Expasy ProtParam [57]. 

### 4.7. Validation of RNA Sequencing

The top differentially expressed potato genes identified in the RNA sequencing analysis all produced multiple transcripts that were difficult to distinguish with quantitative RT-PCR Therefore only *A. solani* genes were chosen for the analysis. We have selected both up- and downregulated transcripts that were found across all the different sampling points or at specific time points only. The leaf disc samples used for the validation experiment were different biological replicates of the ones used for the RNA sequencing analysis. The total RNA was extracted with RNeasy Plant Mini kit (Qiagen, Hilden, Germany) and treated with TURBO DNase (Invitrogen, Vilnius, Lithuania) according to the manufacturers’ protocols. The first strand cDNA was synthesized by oligo (dT) priming using Invitrogen SuperScript III Synthesis SuperMix for qRT-PCR kit (Invitrogen, Carlsbad, CA, USA). Quantitative real time PCR (qRT-PCR) was performed using PowerUp SYBR Green (Applied Biosystems, Vilnius, Lithuania) as the fluorescent dye and primers presented in supplementary Table S1. The β tubulin gene from *A. solani* was used as a constitutively expressed endogenous control [58]. The results of the qRT-PCR assays were analyzed using the modified Delta-Delta Ct method as described in AO Avrova, E Venter, PRJ Birch and SC Whisson [59]. The obtained relative expression values were log2 transformed and their correlation to the log2 fold change values from RNA sequencing experiment was analyzed by Pearson correlation coefficient using GraphPad Prism version 9.2.0 for Windows (GraphPad Software, San Diego, CA, USA).

## 5. Conclusions

Early blight caused by *Alternaria solani* poses an increasing problem in potato production. However, we lack an understanding of defense and resistance mechanisms in potatoes. Here, we have presented an RNA sequencing-based transcriptome data set and analysis of potato cultivar Désirée infected with *A. solani* of time points before penetration, at the start of penetration and when necrosis of groups of plant cells occur. The majority of the differentially expressed plant transcripts identified were time point, and thus likely infection stage-specific, presenting an interesting data set for further mining of the potential resistance and susceptibility factors that might give clues for future improvement of host plant resistance to early blight in potato. Additionally, the differentially expressed *A. solani* transcripts detected in this study present interesting candidates for further validation studies on their role in pathogenicity.

## Figures and Tables

**Table 1:** Transcriptome statistics of *S. tuberosum* and *A. solani* during infection. Total reads (million), percentage of reads mapping to potato reference genome and percentage of reads unmapped to potato genome mapping to *A. solani* reference genome for all *A. solani* inoculated samples per time point, average of biological replicates given.

	Time Point (Hours Post Inoculation)				
	1	6	12	24	48
Total reads (Million)	27.33	28.23	31.06	29.55	35.17
Mapping to *S. tuberosum* reference genome (%)	87.2	88.5	86.6	86.6	88.9
Unmapped to *S. tuberosum* mapping to *A. solani* reference genome (%)	1.64	4.17	1.63	0.27	0.05

**Table 2:** Top 10 differentially expressed potato transcripts in *A. solani* inoculated samples at 1 hpi. Transcript ID, log2 Fold change, and the gene descriptions are displayed.

Transcript ID	log2 Fold Change	Gene Description
PGSC0003DMT400020751	7.74	Hydrolase
PGSC0003DMT400029007	7.65	ATPase
PGSC0003DMT400021742	7.65	Mutt domain protein
PGSC0003DMT400078504	−7.56	ALG2-interacting protein X
PGSC0003DMT400026165	−7.57	Stress regulated protein
PGSC0003DMT400076667	−7.66	WRKY transcription factor
PGSC0003DMT400006755	−7.81	Protein disulfide isomerase
PGSC0003DMT400061097	−7.89	Phosphomethylpyrimidine kinase
PGSC0003DMT400079490	−8.45	Chromatin remodeling complex subunit
PGSC0003DMT400073533	−8.57	Sugar transporter

**Table 3:** Differentially expressed *A. solani* transcripts overlapping in *A. solani* inoculated samples at 6, 12, 24 and 48 hpi. Transcript ID, log2 fold change, and the gene descriptions based on the RATtk v1.073 and BLAST analysis are displayed.

Transcript ID	Log2 Fold Change			Gene Description RASTtk-v1.073		Gene Description *Alternaria alternata*
	6 hpi	12 hpi	24 hpi	48 hpi		
mRNA_3291	−2.68	−8.17	−4.52	−4.52	Pectate lyase (EC 4.2.2.2)	pectate lyase precursor
mRNA_8720	−2.54	−4.34	−8.14	−6.31	Inositol-1-phosphate synthase (EC 5.5.1.4)	Myo-inositol-1-phosphate synthase
mRNA_10760	4.86	6.02	5.78	6.74	hypothetical protein	NADP-dependent mannitol dehydrogenase
mRNA_10750	5.62	7.78	7.53	6.69	Lead, cadmium, zinc and mercury transporting ATPase (EC 3.6.3.3) (EC 3.6.3.5)	potassium/sodium eff

**Table 4:** Top 5 up- and downregulated *A. solani* transcripts at 6, 12, 24 and 48 hpi compared to 1 hpi. Transcript ID, log2 Fold change, and the gene descriptions based on the RATtk v1.073 and BLAST analysis are displayed. Predicted signal peptide determined by SignalP 5.0 with likelihood > 0.9, and predicted non-cytoplasmic domain based on InterPro analysis [12]. x indicates presence.

Time Point	Transcript ID	Log^2^ Fold Change	Gene Description RASTtk-v1.073	Gene Description *Alternaria alternata*	Predicted Signal Peptide/Non-Cytoplamic Domain
6 hpi	mRNA_9018	7.98	Aldehyde dehydrogenase (EC 1.2.1.3)	aldehyde dehydrogenase	
	mRNA_10227	6.96	Acetyl-CoA hydrolase/transferase family protein	hypothetical protein	
	mRNA_11966	6.60	hypothetical protein	hypothetical protein	x/x
	mRNA_9568	6.57	hypothetical protein	hypothetical protein	
	mRNA_5212	6.51	Malate dehydrogenase (EC 1.1.1.37)	malate dehydrogenase-like protein	
	mRNA_9008	−4.90	hypothetical protein	hypothetical protein	x/x
	mRNA_914	−5.75	hypothetical protein	S-adenosyl-L-methionine-dependent methyltransferase	
	mRNA_4527	−5.99	hypothetical protein	hypothetical protein	
	mRNA_9891	−6.42	monooxygenase FAD-binding protein	monooxygenase	
	mRNA_8569	−6.92	hypothetical protein		x/x
12 hpi	mRNA_9018	10.21	Aldehyde dehydrogenase (EC 1.2.1.3)	aldehyde dehydrogenase	
	mRNA_29	8.93	Trehalase (EC 3.2.1.28)	trehalase	x/x
	mRNA_10328	8.82	hypothetical protein	hypothetical protein	
	mRNA_11100	8.70	hypothetical protein	opsin-1	
	mRNA_5135	8.70	hypothetical protein	GroES-like protein	
	mRNA_4571	−5.43	hypothetical protein	hypothetical protein	x/x
	mRNA_10884	−5.46	hypothetical protein	hypothetical protein	
	mRNA_8569	−5.79	hypothetical protein		x/x
	mRNA_714	−5.97	Glycerol-3-phosphate dehydrogenase [NAD(P)%2B] (EC 1.1.1.94)	NAD-dependent glycerol-3-phosphate dehydrogenase	
	mRNA_3291	−8.17	Pectate lyase (EC 4.2.2.2)	pectate lyase precursor	x/x
24 hpi	mRNA_6735	10.92	hypothetical protein	heme oxygenase-like protein	
	mRNA_6435	9.47	hypothetical protein	hypothetical protein	
	mRNA_7342	9.07	hypothetical protein	DUF1761-domain-containing protein	
	mRNA_5135	8.90	hypothetical protein	GroES-like protein	
	mRNA_2512	8.14	Oxidoreductase, GMC family	glucose-methanol-choline oxidoreductase-like protein	x/x
	mRNA_9110	−5.20	Adenosylhomocysteinase (EC 3.3.1.1)	S-adenosyl-L-homocysteine hydrolase	
	mRNA_8537	−6.08	hypothetical protein	general substrate transporter	
	mRNA_3291	−4.52	Pectate lyase (EC 4.2.2.2)	pectate lyase precursor	x/x
	mRNA_4806	−6.74	cell wall surface anchor family protein		
	mRNA_8720	−8.14	Inositol-1-phosphate synthase (EC 5.5.1.4)	Myo-inositol-1-phosphate synthase	
48 hpi	mRNA_10760	6.74	hypothetical protein	NADP-dependent mannitol dehydrogenase	
	mRNA_10750	6.69	Lead, cadmium, zinc and mercury transporting ATPase (EC 3.6.3.3) (EC 3.6.3.5)	potassium/sodium eff	
	mRNA_10227	6.53	Acetyl-CoA hydrolase/transferase family protein	hypothetical protein	
	mRNA_5802	5.76	hypothetical protein	peptidyl-prolyl cis-trans isomeras-like protein	
	mRNA_3291	−4.52	Pectate lyase (EC 4.2.2.2)	pectate lyase precursor	x/x
	mRNA_4806	−4.94	cell wall surface anchor family protein		
	mRNA_8720	−6.31	Inositol-1-phosphate synthase (EC 5.5.1.4)	Myo-inositol-1-phosphate synthase	

## Data Availability

All raw sequencing data in this study have been deposited in National Center for Biotechnology Information (NCBI) under BioProject accession number PRJNA755645.

## Supplementary Materials

The following are available online at https://www.mdpi.com/article/10.3390/plants10102212/s1, supplementary File S1: Output from differential plant transcript expression analysis at 1, 6, 12, 24, and 48 h between *A. solani* inoculated and mock inoculated samples. supplementary File S2: Output from differential *A. solani* transcript expression analysis of *A. solani* inoculated samples at 6, 12, 24, and 48 h compared to 1 hpi, supplementary Figure S1: Correlation between RNA sequencing and qRT-PCR gene expression data, supplementary Table S1: Primer pair sequences used for qRT-PCR validation of RNA sequencing.
