# Supplementary material for: Transcriptome Analysis of Potato Infected with the Necrotrophic Pathogen Alternaria solani
Source: Plants (Basel). 2021 Oct 18;10(10):2212. doi: 10.3390/plants10102212 (PMC8539873; doi:10.3390/plants10102212)
Supplement: Supplementary file 1 [file plants-10-02212-s001.zip › plants-1363663-supplementary/supplemental proofs-1363663 Alternaria ms_/Supplementary file 3.pdf]

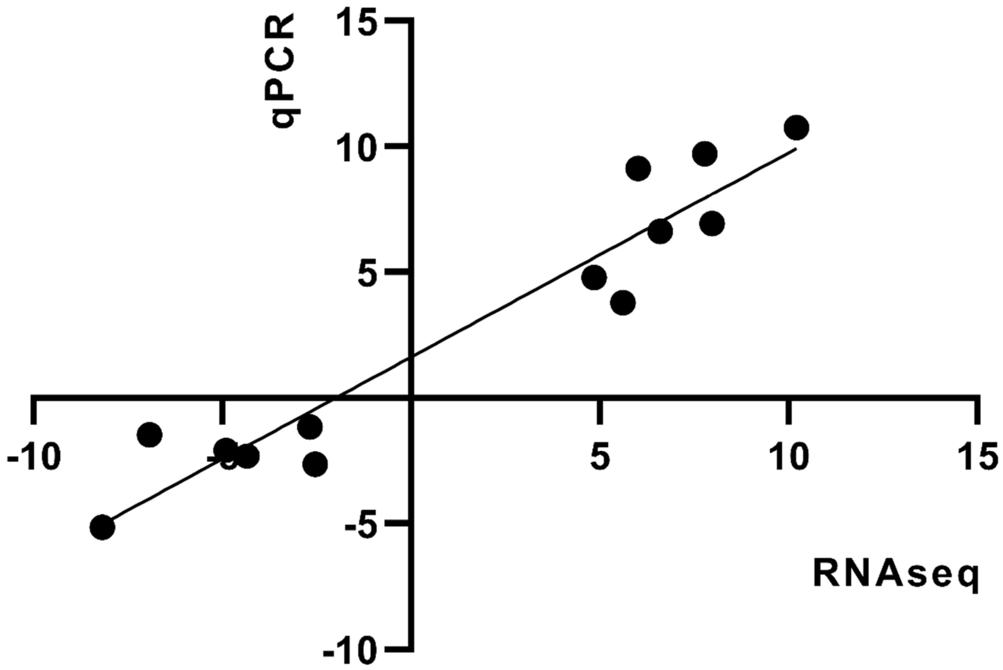

Supplementary Figure S1. Correlation between RNA sequencing and qRT-PCR gene expression data. The top differentially expressed transcripts found in RNA sequencing analysis (both up- and down-regulated genes) were selected. Tubulin β gene was used as a reference gene for the qRT-PCR expression analysis and the presented fold change was calculated relative to the expression at 1hpi. The correlation between the two data sets is presented as linear regression. The R2 value was calculated to be 0.9162 and the Pearson correlation coefficient 0.9572. P value <0.0001.
