# Supplementary material for: Transcriptome Analysis of Potato Infected with the Necrotrophic Pathogen Alternaria solani
Source: Plants (Basel). 2021 Oct 18;10(10):2212. doi: 10.3390/plants10102212 (PMC8539873; doi:10.3390/plants10102212)
Supplement: Supplementary file 1 [file plants-10-02212-s001.zip › plants-1363663-supplementary/supplemental proofs-1363663 Alternaria ms_/Supplementary Table 1.pdf]

Supplementary Table S1. Primer pair sequences used for qRT-PCR validation of RNA sequencing.

Transcript ID/gene	Primer name	Primer sequence
mRNA_3291	As_3291_F	GTTCCGTCACCTACAAGGCA
	As_3291_R	CAGTGGACACCCTCGATCTG
mRNA_8720	As_8720_F2	GTGAAGACGACTCCCAAGGA
	As_8720_R2	AACGTCGGTGGTACGGTAAG
mRNA_10760	As_10760_F2	ACCTCGCCATCACCTACAAC
	As_10760_R2	TGAGCGTACTCGTTGACCTG
mRNA_10750	As_10750_F	AACGATACTTGCGACGTGGT
	As_10750_R	CAACCAGTCCAGCCATGACA
mRNA_9018	As_9018_F2	AACCCAAGTACCGAGGAGGT
	As_9018_R2	TCTGTTCTCGGGTGTCTCCT
mRNA_11966	As_11966_F2	ATCTCTCTGGCGCAATTCAT
	As_11966_R2	CAAGACTGCCATTGACGAGA
mRNA_29	As_29_F2	CATTAATGGCAGCGTGATTG
	As_29_R2	AAATGCTGCGAGCACTTCTT
mRNA_9008	As_9008_F2	CTGCCTCCATTACGTTCGTT
	As_9008_R2	CTCGGTCAATGCTGAGTCAA
mRNA_8569	As_8569_F2	GGAGCCATCGAGATCCTACA
	As_8569_R2	CGCATTCCAGTAGCAGTTCA
β tubulin	As_B tubulin_F	GCTCCCACTCCTTCCGCGC
	As_B tubulin_R	GGAGGTGGAGTTACCGACAA
